# Perceptions and Practices of Japanese Nurses Regarding Tobacco Intervention for Cancer Patients

**DOI:** 10.2188/jea.JE20110008

**Published:** 2011-09-05

**Authors:** Chie Taniguchi, Fukuyo Hibino, Etsuko Kawaguchi, Misae Maruguchi, Naomi Tokunaga, Hideo Saka, Isao Oze, Hidemi Ito, Akio Hiraki, Sumie Nakamura, Hideo Tanaka

**Affiliations:** 1 Department of Nursing, National Hospital Organization, Nagoya Medical Center, Nagoya, Japan; 2 Department of Nursing, National Cancer Center Central Hospital, Tokyo, Japan; 3 Department of Nursing, National Hospital Organization, Osaka Medical Center Hospital, Osaka, Japan; 4 Department of Respiratory Medicine, National Hospital Organization, Nagoya Medical Center, Nagoya, Japan; 5 Division of Epidemiology and Prevention, Aichi Cancer Center Research Institute, Nagoya, Japan; 6 Department of Nursery, Aichi Cancer Center Central Hospital, Nagoya, Japan

**Keywords:** smoking cessation, intervention, nurses, perception

## Abstract

**Background:**

We investigated the perceptions and practices regarding tobacco intervention among nurses, as improvement of such practices is important for the management of patients who smoke.

**Methods:**

Self-administered questionnaires were delivered by hospital administrative sections for nursing staff to 2676 nurses who were working in 3 cancer hospitals and 3 general hospitals. Of these, 2215 (82.8%) responded.

**Results:**

Most nurses strongly agreed that cancer patients who had preoperative or early-clinical-stage cancer but continued to smoke should be offered a tobacco use intervention. In contrast, they felt less need to provide tobacco use intervention to patients with incurable cancer who smoked. Most nurses felt that although they assessed and documented the tobacco status of cancer patients, they were not successful in providing cessation advice, assessing patient readiness to quit, and providing individualized information on the harmful effects of tobacco use. In multivariate analysis, nurses who received instruction on smoking cessation programs during nursing school were more likely to give cessation advice (odds ratio, 1.61; 95% confidence interval, 1.15–2.26), assess readiness to quit (1.73, 1.09–2.75), and offer individualized explanations of the harmful effects of tobacco (1.94, 1.39–2.69), as compared with nurses who had not received such instruction.

**Conclusions:**

The perceptions of Japanese nurses regarding tobacco intervention for cancer patients differed greatly by patient treatment status and prognosis. The findings highlight the importance of offering appropriate instruction on smoking cessation to students in nursing schools in Japan.

## INTRODUCTION

Smoking cessation reduces the risk of developing tobacco-related cancer.^1^ In addition, preoperative abstinence from cigarette smoking can reduce pulmonary and wound-related complications among patients with cancer^2^^–^^4^ and patients undergoing orthopedic surgery.^5^ Smoking cessation also prevents recurrence in patients with a potentially curable tobacco-related cancer,^6^^,^^7^ reduces the risk of developing a secondary tobacco-related cancer,^8^^,^^9^ decreases the risk of treatment side effects,^10^ and improves cancer survival.^11^ These findings demonstrate the importance of tobacco intervention practices for cancer patients who smoke.

During screening, diagnosis, treatment, rehabilitation, and supportive care, nurses have many opportunities to intervene with smokers and recent quitters at risk for relapse, and evidence shows that nurses can provide effective tobacco cessation interventions.^12^^,^^13^ However, attitudes toward such interventions might differ according to the characteristics of nurses and patient health status. Little is known about the perceptions and practices of nurses regarding tobacco intervention for cancer patients in Japan. Thus, we administered a questionnaire survey to examine the perceptions and practices of Japanese nurses working in cancer hospitals and general hospitals regarding tobacco intervention for cancer patients.

## METHODS

### Study subjects

We selected 6 hospitals from among cancer hospitals and general hospitals in Japan. Three are classified as designated cancer hospitals by the Japanese Ministry of Health, Labour and Welfare, ie, they have more than 399 beds and more than 84% of inpatients are cancer inpatients (National Cancer Center Central Hospital, Tokyo; Aichi Cancer Center Central Hospital, Aichi; Kyusyu Cancer Center Hospital, Fukuoka). The other 3 are general hospitals with more than 649 beds and in which 20% to 35% of inpatients are cancer inpatients (Iwate Prefectural Hospital, Iwate; Nagoya Medical Center Hospital, Aichi; Osaka Medical Center Hospital, Osaka). There were 2782 nurses working at the 6 selected hospitals in April 2008. We excluded nurses who were absent for 1 month or longer (eg, due to pregnancy or illness), and the remaining 2676 nurses were eligible to participate.

### Questionnaire survey

We mailed a self-administered questionnaire to the administrative section for nursing staff in each of the 6 hospitals. The administrative section then delivered the questionnaire with a cover letter and a return envelope to the study subjects and asked that they return it anonymously to the administrative section within 2 weeks. The cover letter explained to the nurses that their participation in this study was completely voluntary. To maintain subject autonomy, we did not send reminder letters. This study was approved by the Institutional Review Board of Nagoya Medical Center.

### Questionnaire items

The questionnaire items comprised subject demographics, perceptions toward tobacco use interventions, and recent 3-year practice in tobacco use interventions. The nurses were asked about their perceptions toward tobacco use interventions for 5 categories of hypothetical cancer patients, which were based on patient physical condition, treatment modality, and/or prognosis: (a) preoperative patients, (b) postoperative patients with early-clinical-stage cancer, (c) postoperative patients who received chemoradiotherapy and have an expected survival period of approximately 3 years, (d) postoperative patients who have clinically advanced cancer but are now free from symptoms and have an expected survival time of 1 year, and (e) patients with a terminal prognosis receiving palliative care. In each of the 5 categories, we established 2 subcategories (5 × 2) according to the type of cancer: tobacco-related cancers (head and neck, esophagus, and lung) and other cancers. For the 10 categories of patients, the nurses’ perception of the importance of tobacco intervention was assessed using 5 response categories, ranging from “strongly agree” to “strongly disagree”.

The nurses were asked about the frequency of their involvement in tobacco assessment and interventions in practice using a 4-point scale ranging from “almost always” to “never or rarely”. The questionnaire items included were: (a) assessed and documented tobacco use, (b) provided cessation advice, (c) assessed readiness to quit, (d) provided individualized information about the harmful effects of tobacco use, and (e) made arrangements for enrollment in a smoking cessation program.

The questionnaire included the respondent’s demographic, professional, and institutional characteristics, and his/her own smoking status. We also asked whether they had received instruction on smoking cessation programs in nursing school.

### Assessment and statistical methods

**Figure A1.**
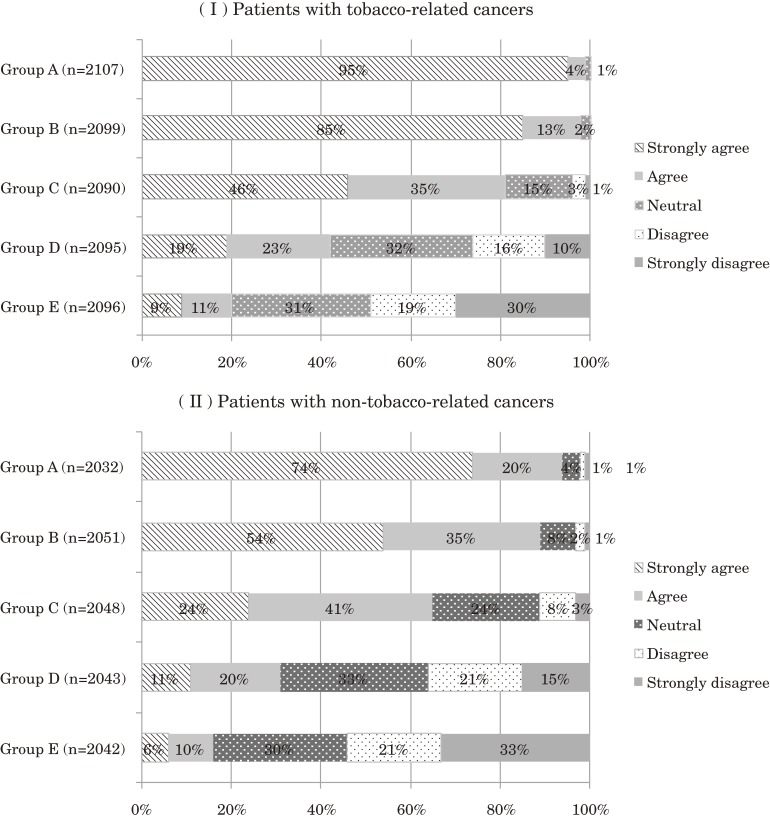
Responses of nurses regarding the need for tobacco intervention in cancer patients, by cancer type and state of health. Group A: Preoperative patients. Group B: Postoperative patients with early-clinical-stage cancer. Group C: Patients who received chemoradiotherapy and have an expected survival time of approximately 3 years. Group D: Postoperative patients who have clinically advanced cancer but are now free from symptoms and have an expected survival time of 1 year. Group E: Patients with a terminal prognosis receiving palliative care.

The summary statistics of perceptions toward tobacco use interventions indicate the proportion of those who indicated that they strongly agreed with a questionnaire item, as we felt that this proportion best reflected the distribution of perception in the 5-part response categories (Figure A1). The summary statistics of tobacco assessment and interventions were calculated using the proportion of those who responded “almost always” to each questionnaire item. The chi-square test was used to compare summary statistics between strata. To elucidate factors associated with perceptions and practices of tobacco use assessment or interventions for cancer patients who smoke, we performed multivariate logistic regression analysis using the following independent variables: age 20 to 29 (yes/no), working in inpatient care (yes/no), working in a surgical division (yes/no), received any academic certification (yes/no), working in a designated cancer hospital (yes/no), and received instruction on smoking cessation programs during nursing school (yes/no). All analyses were performed using STATA version 10 (STATA Corp, College Station, TX, USA).

## RESULTS

The response rates at the 6 hospitals were as follows. Designated cancer hospitals—National Cancer Center Central Hospital (Tokyo), 85% (397/468); Aichi Cancer Center Central Hospital (Aichi), 84% (288/342); Kyusyu Cancer Center (Fukuoka), 89% (233/261). General hospitals—Iwate Prefectural Hospital (Iwate), 76% (392/517); Nagoya Medical Center Hospital (Aichi), 86% (431/499); and Osaka Medical Center Hospital (Osaka), 80% (474/589).

Table 1 shows the characteristics of the 2115 respondents: 41% of respondents worked at a designated cancer hospital, 96% were female, and just over half (51%) were aged 20 to 29 years. Seventy-three percent had received a 3-year nursing degree, 45% had worked for less than 6 years as a nurse, 74% were currently working in an inpatient care setting, and 83% were staff nurses. Only 8% reported current smoking; 12% were former smokers.

**Table 1.** Characteristics of the study subjects (*n* = 2215)

Characteristic	*n*	%
Designated cancer hospital	918	41
General hospital	1297	59

Female	2128	96
Age, years		
20–29	1137	51
30–39	699	32
40+	376	17
Length of nursing education, years		
2	184	8
3	1628	73
4	348	16
Master’s degree	15	<1

Length of employment as a nurse, years		
<3	581	26
3–5	418	19
6–9	378	17
10–15	409	18
≥16	426	19
Current work setting		
Inpatient care	1642	74
Outpatient care	204	9
Operating room/intensive care unit	295	13
Other	65	3
Primary position		
Staff nurse	1834	83
Head nurse	229	10
Supervising nurse	93	4
Assistant director/director	10	<1
Certified by Japan Nursing Association	51	2
Certified by other academic society	30	1

Smoking status		
Current smoker	170	8
Ex-smoker	275	12
Never smoker	1731	78

**Figure 1.**
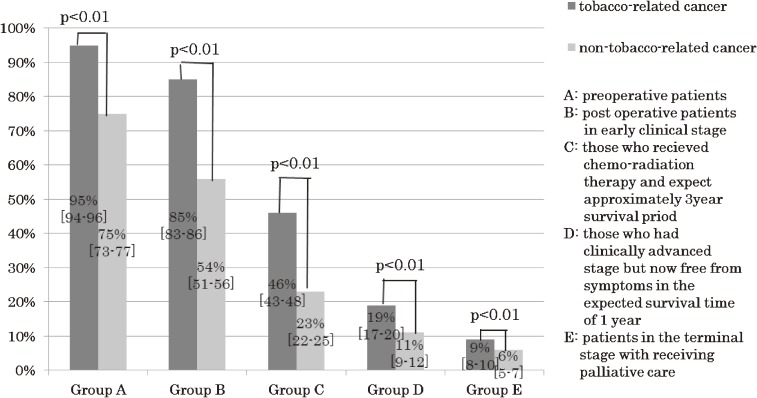
Proportion of study subjects who strongly agreed with providing tobacco intervention to cancer patients in various states of health (A–E).

The nurses’ perceptions toward tobacco use intervention varied widely with regard to the physical condition and prognosis of the cancer patients (Figure 1). Most nurses strongly agreed that tobacco use intervention should be provided to currently smoking cancer patients who were in a preoperative stage or had early-clinical-stage cancer. In contrast, they felt less need to provide intervention to incurable cancer patients who smoke. The subjects felt that the need for tobacco use intervention was significantly higher in patients with tobacco-related cancers than in those with non-tobacco-related cancers in all 5 categories (*P* < 0.01).

The proportions of responses in each of the 5 categories of tobacco intervention perception are shown in Supplemental Figure A1. The proportion of nurses who strongly agreed or agreed with the need for tobacco use intervention declined with deteriorating patient health. In multivariate analysis, the nurses working in designated cancer hospitals had a significantly more positive perception of tobacco intervention for preoperative cancer patients than did nurses working in general hospitals (odds ratio [OR] 2.67, 95% confidence interval [CI] 1.60–4.45 for patients with tobacco-related cancers; OR 1.79, 95% CI 1.43–2.25 for patients with non-tobacco-related cancers). In contrast, nurses working in designated cancer hospitals had a significantly more negative perception of tobacco intervention for patients with a terminal prognosis receiving palliative care (OR 0.66, 95% CI 0.51–0.84 for patients with tobacco-related cancers; OR 0.57, 95% CI 0.40–0.81 for patients with non-tobacco-related cancers).

**Figure 2.**
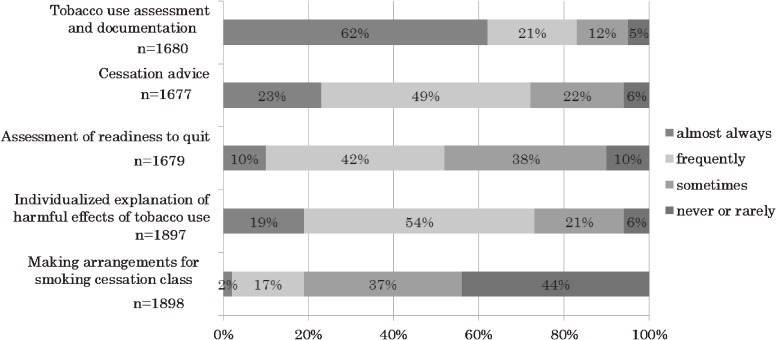
Frequency of performing tobacco use assessment and intervention during a recent 3-year period for currently smoking patients with cancer.

The frequency of involvement in tobacco assessment and intervention varied widely, as shown in Figure 2: 62% of nurses responded that they “almost always” assessed and documented tobacco use, whereas only 10% indicated that they “almost always” assessed readiness to quit in cancer patients. Cessation advice to cancer patients who smoke was “almost always” or “frequently” provided by 72% of the respondents, whereas only 19% of them made arrangements for enrolling patients in a smoking cessation program (Figure 2).

The frequency of tobacco use assessment and documentation significantly differed according to respondent age, length of nursing education, current work setting (inpatient care/other), nursing certification status, and type of hospital (cancer hospital/general hospital) (Table 2). Current work setting (surgical division/other), type of hospital, and history of receiving instruction on smoking cessation programs in nursing school were significantly associated with the frequency of providing cessation advice. Assessment of readiness to quit and providing individualized explanation of the harmful effects of tobacco use were significantly associated with the nurses’ current work setting, certification status, type of hospital, and history of receiving instruction in smoking cessation programs. There was no significant difference in the frequency of tobacco assessment or intervention with regard to respondent smoking status (Table 2).

**Table 2.** Proportions of nurses who, during their most recent 3 years of practice, almost always performed tobacco use assessments or interventions for currently smoking cancer patients, by characteristics of nurses

Nurse characteristic	Tobacco use assessment and documentation				Cessation advice			Assessment of readiness to quit			Individualized explanation of harmful effects of tobacco use		
			
	*n*	%	95% CI	*P* value	%	95% CI	*P* value	%	95% CI	*P* value	%	95% CI	*P* value
Age 20–29													
Yes	1137	65	61.4–67.8	**0.01**	24	20.8–26.4	0.69	9	7.0–10.7	0.14	19	16.1–20.9	0.19
No	1075	59	55.1–62.0		23	19.9–25.7		11	8.8–13.1		26	18.2–23.5	

Length of nursing education													
≥4 years	363	70	64.6–75.5	**<0.01**	24	19.3–29.5	0.61	10	7.5–15.0	0.37	20	15.8–24.9	0.71
<4 years	1845	60	57.5–62.6		23	20.7–25.2		11	8.0–11.0		19	17.5–21.4	

Current work setting													
Inpatient care	1642	64	61.1–66.3	**<0.01**	24	21.8–26.5	0.12	9	7.9–11.1	0.28	20	17.7–21.7	0.99
Other	564	55	49.8–60.2		20	16.0–24.5		11	8.1–14.7		20	15.8–23.6	

Current work setting													
Surgical division	883	63	58.9–67.7	0.5	28	24.9–33.2	**<0.01**	11	7.2–12.6	0.48	24	20.3–27.6	**<0.01**
Other	1335	61	57.9–63.8		21	18.5–23.4		10	7.9–11.5		18	15.4–19.8	

Any academic certification													
Yes	126	52	41.3–61.8	**0.04**	31	21.1–40.0	0.08	20	11.8–28.2	**<0.01**	29	20.0–37.4	**0.01**
No	2074	62	60.0–64.8		23	20.7–24.9		9	8.9–10.8		19	17.1–20.8	

Workplace													
Designated cancer ​ hospital	918	73	69.9–76.4	**<0.01**	35	31.7–38.8	**<0.01**	15	12.3–17.6	**<0.01**	28	24.8–31.1	**<0.01**
General hospital	1297	53	50.1–56.4		14	12.1–16.6		6	4.6–7.6		14	11.7–15.7	

Received instruction^a^													
Yes	346	68	62.0–73.8	0.05	31	25.7–37.4	**<0.01**	13	9.6–18.3	**0.04**	27	22.2–32.6	**<0.01**
No	1838	61	58.6–63.6		22	19.7–23.9		9	7.6–10.6		18	16.2–19.9	
Attended lecture^b^													
Yes	210	69	61.3–76.6	0.05	23	16.0–29.9	0.92	14	8.1–19.6	0.12	25	18.8–32.1	0.06
No	1965	61	59.0–63.9		23	21.3–25.6		10	8.1–11.1		19	17.0–20.7	

Smoking status													
Never	1731	62	59.3–64.6	0.62	23	20.8–25.3	0.6	10	8.2–11.5	0.65	19	17.3–21.3	0.58
Current or ex-smoker	445	61	55.4–65.7		24	19.6–28.6		9	6.1–12.2		20	16.3–24.3	

^a^Received instruction on smoking cessation programs at his/her nursing school.

^b^Attended a lecture on smoking cessation intervention at his/her hospital.

95% CI: 95% confidence interval.

In multivariate analysis, the current work setting of inpatient care was significantly associated with performing tobacco use assessments and documentation (OR 1.57, 95% CI 1.14–2.16; Table 3). Current work in a surgical setting was significantly associated with providing cessation advice (OR 1.83, 95% CI 1.40–2.39) and providing individualized explanation of the harmful effects of tobacco use (OR 1.58, 95% CI 1.21–2.05). Nurses with an academic certification were significantly more likely to assess readiness to quit than those without such certification (OR 2.33, 95% CI 1.29–4.21). All 4 tobacco intervention practices were significantly more frequent among nurses working in a designated cancer hospital than among those in general hospitals. Nurses who received instruction on smoking cessation programs in their nursing school were significantly more likely to provide cessation advice (OR 1.61, 95% CI 1.15–2.26), assessment of readiness to quit (OR 1.73, 95% CI 1.09–2.75), and individualized explanation of the harmful effects of tobacco use (OR 1.94, 95% CI 1.39–2.69).

**Table 3.** Factors associated with almost always performing tobacco use assessment and interventions for currently smoking cancer patients during the most recent 3 years of practice in Japanese nurses (multivariate logistic regression)

	Tobacco use assessment and documentation			Cessation advice			Assessment of readiness to quit			Individualized explanation of harmful effects of tobacco use		
				
	OR	(95% CI)	*P* value	OR	(95% CI)	*P* value	OR	(95% CI)	*P* value	OR	(95% CI)	*P* value
Age 20–29 years	1.13	(0.89–1.42)	0.31	0.98	(0.75–1.29)	0.9	0.71	(0.48–1.04)	0.08	0.78	(0.60–1.03)	0.08
Inpatient care	1.57	(1.14–2.16)	0.006	1.41	(0.94–2.11)	0.1	0.93	(0.56–1.54)	0.78	1.02	(0.70–1.50)	0.9
Surgical division	1.21	(0.96–1.53)	0.11	1.83	(1.40–2.39)	0.000	1.02	(0.70–1.51)	0.89	1.58	(1.21–2.05)	0.001
Any academic certification	0.69	(0.43–1.11)	0.13	1.57	(0.92–2.67)	0.1	2.33	(1.29–4.21)	0.005	1.63	(0.99–2.69)	0.06
Designated cancer hospital	2.36	(1.88–2.95)	0.000	3.49	(2.70–4.51)	0.000	2.71	(1.89–3.88)	0.000	2.58	(2.00–3.32)	0.000
Received instruction^a^	1.18	(0.86–1.62)	0.3	1.61	(1.15–2.26)	0.006	1.73	(1.09–2.75)	0.02	1.94	(1.39–2.69)	0.000

^a^Received instruction on smoking cessation programs at his/her nursing school.

OR: odds ratio, 95% CI: 95% confidence interval.

## DISCUSSION

To our knowledge, there have been no Asian studies of nurses’ perceptions of tobacco intervention for cancer patients who smoke, although the attitudes of people with cancer regarding smoking cessation and the patient education practices of oncology nurses in Japan, Taiwan, and Korea were reported in a small study.^14^ Our study showed that nurses’ perceptions toward tobacco intervention were highly dependent on the health and prognosis of cancer patients and whether their cancer was tobacco-related. The Japanese nurses showed less willingness to provide tobacco intervention for cancer patients with a poor prognosis. In particular, the nurses working in designated cancer hospitals had a significantly more negative perception of tobacco intervention for patients with a terminal prognosis who were receiving palliative care, possibly because they believed that these patients would derive limited benefit from smoking cessation. However, we believe that this attitude is not appropriate because continued smoking reduces treatment effectiveness and results in faster deterioration of health, even in patients with incurable cancer. The present study also showed that the Japanese nurses were less willing to provide tobacco intervention for patients with non-tobacco-related cancers than for those with tobacco-related cancers. This was probably due to the nurses’ incorrect belief that currently smoking patients with non-tobacco-related cancers do not believe that smoking cessation would improve their health and/or they are less motivated than those with tobacco-related cancers to stop smoking. Therefore, we believe that modifying nursing education might change the incorrect attitudes of nurses toward tobacco intervention for cancer patients in Japan.

Regarding tobacco intervention practice, although most nurses assessed and documented the tobacco status of their patients, they did not often provide cessation advice, assess readiness to quit, provide individualized information about the harmful effects of tobacco use, or make arrangements for patients to enroll in a smoking cessation program. Except for assessing and documenting tobacco status, the frequencies of these practices in the present study were lower than those among oncology nurses in the United States assessed in 1998, as indicated by the proportions of nurses reporting a frequency of “every day” or “every week” (provided cessation advice: 23% vs 32%; assessed readiness to quit: 10% vs 38%). However, it should be noted that the response rate in the US survey was only 38%.^15^ The low frequency of making arrangements for cancer patients to enroll in a smoking cessation program in the present study was possibly influenced by the considerable number of patients with limited readiness to quit and low activities of daily living, as well as the limited availability of smoking cessation programs in patients’ areas of residence.

From the perspective of nurses’ behavior regarding tobacco intervention for cancer patients who smoke, an important finding was that these behaviors were positively associated with a history of instruction in smoking cessation programs during nursing school, after adjustment for a number of confounding factors. This finding confirmed the importance of providing instruction on smoking cessation in the standard curriculum of nursing schools in Japan.

One limitation of our study was the representativeness of the sample we obtained. We selected nurses working at 3 designated cancer hospitals and 3 general hospitals. Although their baseline characteristics were well documented, the findings may not be applicable to nurses working in smaller hospitals, as their characteristics might differ from those of our respondents. Our multivariate analysis showed that nurses working in the designated cancer hospitals and those with any academic certification in nursing education or technique were more likely to provide smoking cessation interventions for cancer patients, which suggests that the frequency of smoking cessation intervention by nurses in the present study might be higher than that among Japanese nurses in general. To improve representativeness, we need to perform another survey of nurses stratified by specialty and hospital size. Our study did not assess the tobacco intervention perception and practices of nurses with regard to patients’ readiness to quit and other behavior-related characteristics such as self-efficacy in quitting. To improve the usefulness of the assessment, we need to examine these items on patient smoking-related characteristics in a future study.

In conclusion, we observed that the perceptions of Japanese nurses toward tobacco intervention in cancer patients differed greatly with regard to patient treatment status and prognosis. In addition, the nurses’ tobacco intervention practices were significantly associated with a history of instruction in smoking cessation programs while they were in nursing school. These findings should be useful in improving tobacco intervention attitudes and practices among nurses treating patients with cancer in Japan.
